# Antimicrobial potentials of *Pandanus amaryllifolius* Roxb.: Phytochemical profiling, antioxidant, and molecular docking studies

**DOI:** 10.1371/journal.pone.0305348

**Published:** 2024-08-14

**Authors:** Dwi Kusuma Wahyuni, Gita Aqila Nuha, Tope Gafar Atere, Viol Dhea Kharisma, Vinaya Satyawan Tari, Cici Tya Rahmawati, Ahmad Affan Ali Murtadlo, Alvi Jauharotus Syukriya, Sumrit Wacharasindu, Sehanat Prasongsuk, Hery Purnobasuki

**Affiliations:** 1 Department of Biology, Faculty of Science and Technology, Universitas Airlangga Surabaya, Surabaya, East Java, Indonesia; 2 Department of Medical Biochemistry, College of Health Sciences, Osun State University, Osogbo, Nigeria; 3 Program in Biotechnology, Faculty of Science, Chulalongkorn University, Bangkok, Thailand; 4 Department of Chemistry, Faculty of Science, Chulalongkorn University, Bangkok, Thailand; 5 Plant and Biomass Utilization Research Unit, Department of Botany, Faculty of Science, Chulalongkorn University, Bangkok, Thailand; Museu Paraense Emilio Goeldi, BRAZIL

## Abstract

The emergence of antimicrobial resistance has led to an urgent need for novel antimicrobial drugs. This study aimed to determine the antioxidant and antimicrobial potentials *in silico* and *in vitro* of *Pandanus amaryllifolius* Roxb. ethanolic extract. The extracts were subjected to gas chromatography-mass spectrometry (GC-MS) analysis to identify the compounds. *In silico* antimicrobial studies were performed to gain insights into the possible mechanism of action of the active compounds as antimicrobials. The antimicrobial activities of the ethanolic extracts were assessed using the agar well diffusion method against the Surabaya strain of *Escherichia coli* and *Staphylococcus aureus*. Antioxidant properties of the extract were done using DPPH (2,2-diphenyl-1-picryl-hydrazyl-hydrate) and ABTS [2,2’-azino-bis (3-ethylbenzthiazoline-6-sulphonic acid)] inhibition assays. The phytochemical screening revealed that the extract has high flavonoids and polyphenols contents. The GC-MS analysis detected the presence of 52 bioactive substances, with *n*-hexadecanoic acid, 9, 12, 15-octadecatrienoic acid, benzofuran 2,3-dihydro-. quinic acid, neophytadiene as major compound. Molecular docking studies showed that these compounds have a high binding affinity towards the target proteins, thereby inhibiting their activities. The ethanolic extract of *P*. *amaryllifolius* Roxb. exhibited antioxidant and antimicrobial activities. The IC_50_ were 11.96 ± 4.01 μg/ml and 26.18 ± 7.44 μg/ml for DPPH and ABTS. The diameters of inhibition zones (DIZ) and percentage of inhibition (PI) were calculated and varied for every single pathogen 16.44 ± 1.21mm/66.76 ± 4.92% (50%) and 21.22 ± 0.11mm/82.49 ± 3.91% (50%) for *E*. *coli* and *S*. *aureus* (DIZ/PI) respectively. Overall, this study provides information on the mechanism responsible for *P*. *amaryllifolius* Roxb. extract as a natural antimicrobe and lays the foundation for further studies to isolate and characterize the active compounds as antimicrobial candidates.

## Introduction

Antimicrobial resistance (AMR) severely impacts the foundation of contemporary medicine and the viability of an efficient, worldwide healthcare response to the persistent threat posed by infectious diseases [1–3]. The quest for novel and potent antimicrobial drugs is urgently required since antimicrobial resistance has turned into a worldwide health problem.

With the frustratingly slow development of new medications and pharmaceutical company investment, the abuse of antimicrobials like antibiotics has become a significant problem for both medicine and agriculture [4]. By 2050, according to O’Neill (2016), AMR will lead to death of 10 million population per year surpassing cancer as the leading cause of death. This unsettling prediction and current trend in AMR has motivated researchers to isolate and discover new bioactive compounds from plants that targeted against microbial resistance [5] also notably given that around 50% of existing pharmaceuticals and nutraceuticals are naturally derived products [6, 7].

The historic usage of plants to cure a variety of illnesses, including infectious diseases, and their potential as a source of novel antimicrobial agents [8]. Moreover, chemically complex substances have excellent therapeutic potential since they have less adverse effects than manufactured medications and also have a low likelihood of acquiring resistance [9]. Around 1,340 floras have been identified for specific antibacterial properties, and >30,000 antimicrobial chemicals have been extracted from various plant species [6]. In the battle against AMR, herbal remedies have proven to be an effective tool that may be used alone or in conjunction with existing antibiotic strategies [4]. *Pandanus amaryllifolius* Roxb. ex Lindl. is indigeneous and widely available in Indonesia. Using indigeneous plants for extraction of essentials for betterment of lives is the sustainable approach to achieve sustainable development goals (SDGs) [10].

Many substances, including alkaloids, phenols, spermidine, rutin, quercetin, tocopherol, and carotenoids, have been identified as being present in plants and contributing to their antibacterial potentials [6, 11]. The antibacterial activities of *Discopodium penninervium* Hochst., *Lippia adoensis* Hochst., *Polysphaeria aethiopica* Verdc., *Euphorbia depauperata* Hochst., *Cucumis pustulatus* Hook.f., *Sonchus arvensis* L., *Pluchea indica* L., *Cosmos caudatus* L., *Achillea millefolia* L., *Pterocarpus macrocarpus* Kurz., and *Rumex abyssinicus* Jacq. are due to the presence of alkaloids, polyphenols, tannins, terpenoids, flavonoids, cardiac glycoside and saponins [2, 3, 12–15].

A plant from the Pandanaceae family that is mostly found in Southeast Asian countries, Pandan has been utilized for traditional medicine and ethnobotanical products [16]. Because of their distinctive and pleasant scent, pandan leaves are frequently used in Southeast Asia to flavor a variety of foods, including baked goods, desserts, and even home cooking. The only *P*. *amaryllifolius* Roxb. species with fragrant leaves is remaining to the chemical 2-acetyl-1-pyrroline (2AP), which is responsible for the perfume. Several studies have shown that *P*. *amaryllifolius* Roxb. is a great source of phenolic and flavonoid chemicals. Several studies have found that the leaves and roots of *P*. *amaryllifolius* Roxb. contain bioactive substances such phenolic compounds and flavonoids, which function as antioxidants and may scavenge free superoxide radicals [16, 17].

In an effort to discover and explore the bioactivity compounds which can be antimicrobial agent candidate, there has been an increasing interest in examining the possible antibacterial activity of *P*. *amaryllifolius* Roxb. and its components [16, 18]. This present study intended to explore the antioxidant and antimicrobial potential of *P*. *amaryllifolius* Roxb. ethanolic extract by evaluating its activity *in silico* and *in vitro* methods. The findings could provide valuable outcome for understanding the pharmaceutical potential of *P*. *amaryllifolius* Roxb. as a source of new antioxidant and aid in the development of innovative therapies for the treatment of infectious diseases. Moreover, this study is well contributing to about 5 Sustainable Development Goals (SDGs 17): viz. good health and well-being, sustainable cities and communities, quality education, life on land, and responsible consumption and production etc. designed and adopted to serve as a *"shared blueprint for peace and prosperity for people and the planet*, *now and into the future*.*"* in 2015.

## Materials and methods

### Collection and authentication of plant material

*Pandanus amaryllifolius* Roxb. was collected from Taman Husada Graha Famili (Plant Medicinal Garden) Surabaya, East Java, Indonesia (7º18’12.2”S 112º41’12.7”7E). The healthy and green leaves sample without indications of insect or microbial damage were collected form the site. The sample plant material was authenticated by the Purwodadi Botanical Garden (Indonesian Institute of Sciences, Jakarta, Indonesia). The voucher specimen was placed in the Plant Systematics Laboratory, Department of Biology, Faculty Science and Technology Universitas Airlangga, Indonesia with reference No. PA.0116022023.

### Sample extraction

The leaves of *P*. *amaryllifolius* Roxb. were allowed to dry in open air and then ground into a powder with electric grinder and sieved by using 60-mesh size sieves. Each 100 g of powder was separately soaked with ethanol for 24 hrs at room temperature (28±2°C), same procedure was repeated thrice subsequently, followed by filtration with filter paper (pore diameter 110 mm); Merck KGaA, Darmstadt, Germany, and the filtrate obtained was evaporated in a rotary evaporator at 60°C to acquire crude extracts. The volume of the extract (w/w) was measured before storage at 4°C in the refrigerator [2, 15].

### Phytochemical profiling by gas chromatography-mass spectrometry (GC-MS)

Gas chromatography-mass spectrometry (GC-MS) analysis was done by using “Agilent Technologies”. The specification of GC-MS was agilent technologies model 19091N-136HP-INNOWax, 5% phenyl methyl silox agilent technologies, Initial temperature was 150°C held for 2 min, final temperature was 260°C at the rate of 20°C/min, 1 μl of 0.2 g/ml fraction was injected. Temperature of heater was 300°C, pressure was 27, 213psi, column (60mol/L×250μmol/L×0.25μmol/L) and carrier gas (helium, 99.9999% purity, flow rate = 1,7583mL/min; average velocity was 34,171cm/sec). The constituents of compounds were compared with the retention times and mass spectrum of the samples obtained using gas chromatography with the mass spectra from the National Institute of Standards and Technology (NIST) Version 14MS database library [3, 19].

### *In silico* antimicrobial activity

#### Ligand retrieval

Ligand from *P*. *amaryllifolius* Roxb. ethanol leaves in this study refers to the results of GC-MS. GC-MS data showed fifty-two compounds from *P*. *amaryllifolius* Roxb. 3D structure of ligand, Collision induced dissociation (CID), formula, molecular weight (g/mol), and SMILE Canonical obtained from PubChem database (https://pubchem.ncbi.nlm.nih.gov/). Structure optimization and energy minimization of the ligands were performed through Open Babel v2.3.1 to obtain the PDB file [2, 20, 21].

#### Protein preparation

Target proteins from microorganisms for identification of antimicrobial activity through a computational approach consist of *Bacillus subtilis*—FtsZ, *Candida albicans*—acetohydroxyacid synthase (AHAS), *Escherichia coli*—Rhomboid Protease (Rpro), and *Staphylococcus aureus—*Sortase A (SA). The RCSB database (http://www.rcsb.org/pdb/home/home.do) was used for retrieval of the four target proteins with the program database (pdb) file. Water molecules and native ligands are removed from targets through PyMol v2.5 software [3, 22, 23].

#### Drug likeness prediction

The similarity of drug properties from query compounds in this study was identified through drug likeness analysis. Lipinski Rule of Five from SCFBio server (http://www.scfbio-iitd.res.in/software/drugdesign/lipinski.jsp) was used for the drug like-molecule assessment in this study. Parameters including molecular mass, high lipophilicity (LOGP), hydrogen bond acceptors-donors, and molar refractivity are considered as determinants of the drug like-molecule properties of the query compound [24, 25].

#### Virtual screening

The method used to identify activity from query compound by referring to interactions on targets through a computational approach is virtual screening. The type of virtual screening method used in this study is molecular docking. The Docking study mainly aims to identify the antimicrobial potential of *P*. *amaryllifolius* Roxb. Compounds from *P*. *amaryllifolius* Roxb. extract act as ligands and targets are proteins from microbes such as *Bacillus subtilis*–FtsZ, *Candida albicans*–AHAS, *Escherichia coli*–Rhomboid Protease (Rpro), and *Staphylococcus aureus*–Sortase A (SA). PyRx v0.9.9 software was performed to simulate ligand-protein docking [25, 26]. The docking grid in this study consist FtsZ–RCSB ID: 2VAM–*Bacillus substilis* Center (Å) X:28.973 Y:-8.976 Z:-1.975 Dimensions (Å) X:67.136 Y:62.079 Z:71.526, AHAS–RCSB ID: 6DEK–*Candida albicans* Center (Å) X:56.869 Y:246.767 Z:46.576 Dimensions (Å) X:80.320 Y:59.015 Z:74.313, Rpro–RCSB ID: 3ZMI–*Escherichia coli* (Å) X:15.820 Y:-9.206 Z:42.649 Dimensions (Å) X:47.082 Y:51.738 Z:46.252, SA—RCSB ID: 2MLM—*Staphylococcus aureus* (Å) X:16.421 Y:12.281 Z:11.952 Dimensions (Å) X:48.928 Y:53.323 Z:39.169.

#### Ligand-protein interaction

In the present study, the molecular interactions were identified with the help of Discovery Studio 2016 software. Weak bond interactions can be identified in ligand-protein complexes such as hydrogen, van der Waals, hydrophobic, and pi/alkyl. These interactions serve to trigger activity such as an inhibitory response on the target [27, 28].

#### 3D molecular visualization

Molecular complexes exhibited through PyMol v2.5 software with coloring and structural selection methods. Transparent surfaces and sticks are selected for visualization. Color selection refers to the type of atom and 3D representation [22, 29].

### *In vitro* antioxidant activity

#### The DDPH (2,2-diphenyl-1-picryl-hydrazyl-hydrate) inhibition assay

The DPPH ***(2*,*2-diphenyl-1-picryl-hydrazyl-hydrate)*** inhibition assay was carried out in concurrence of Prieto (2012) and Wahyuni et al. (2023) with required modifications. During analysis 100μl samples at different concentrations from 1.075 to 200 μg/ml in ethanol were added to 100 μl DPPH reagent (0.2 mM) and incubated for 30 min at room temperature (28±2°C), theses mixtures are further used as samples [2, 30]. Whereas, Ascorbic acid (reference standard) was positive control in the experiment. The resulting absorbance was measured at wavelength 517 nm by using microplate reader (Thermo Scientific, USA). Furthermore, the percentage of sample inhibition was calculated by using following Formula (1):

%Inhibition=(Acontrol‐Asample)/Acontrol×100% (1)

Where ‘A sample’ is the absorbance of sample (the mixture of DPPH reagent and sample), whereas, ‘A control’ is the absorbance of only reference standard. The percentage of inhibition at all concentrations were plotted and linear regression analysis was carried out for determination of the half-maximal inhibitory concentration (IC_50_) value.

#### The ABTS [2,2’-azino-bis (3-ethylbenzthiazoline-6-sulphonic acid)] inhibition assay

The ABTS ***[2*,*2’-azino-bis (3-ethylbenzthiazoline-6-sulphonic acid)]*** inhibition assay was performed as per the method illustrated by Fu et al. (2022) and Wahyuni et al. (2023). However, the ABTS reagent was prepared by adding 7 mM ABTS solution with 2.4 mM potassium persulphate solution and mixed well, then stored at room temperature (28±2°C) for 12–16 hours in the dark place. After the waiting period, the absorbance of the solution at wavelength 734 nm was measured (stock solution diluted to obtain absorbance between 0.7–0.72). 100 μl sample at different concentrations from 1.075 to 200 μg/ml in ethanol were mixed with 100 μl of ABTS reagent. Whereas, trolox and ascorbic acid were used as positive control (reference standard). After incubating for 5 mins in the dark place at room temperature (28±2°C), the absorbance was assessed at wavelength 734 nm by using microplate reader (Thermo Scientific, USA). The percent inhibition and IC_50_ value were calculated by using same Formula 1 as given above [2, 31].

#### Antimicrobial activity

The antimicrobial activity was analyzed by using agar well diffusion method against selected gram-positive bacteria and gram-negative bacteria viz. *Staphylococcus aureus* and *Escherichia coli* respectively [2, 3]. Both bacteria were isolated from Surabaya City, East Java, Indonesia. Nutrient agar media was used to culture test microorganism. Whereas, potato dextrose agar was used for cultivating the microbes. The 30 mL sterilized agar medium was used to pour into presterile petri plates with 10cm diameter and all plates are allowed to solidify. Then 100 μL of inoculate with optical density (OD) 0.1 from each selected strain was spread on the solidified agar plates carefully with the help of glass spreader. On the other side a stock solution of plant extract was prepared and diluted serially (25% and 50%). The well of 5 mm diameter, was made in solidified medium in petri plates with the help of a cork borer. Whereas 20% of dimethyl sulfoxide (DMSO) and Chloramphenicol were used as negative control, and positive control respectively. All wells were filled with 30 μL of prepared extract and control accordingly in respective agar petry plates. All plates are incubated for 24 hrs at 37°C, and antimicrobial activity was assessed and diameter of the inhibition zone (DIZ) was measured around the wells in the nutrient agar medium. The percentage of inhibition i.e. PI was calculated as by using Formula (2):

PI=Theinhibitionzoneofthesample(cm)/Thezoneofpositivecontrol(cm)×100% (2)

#### Data analysis

Data are given as the mean ± standard deviation. The IC_50_ values for *in vitro* antioxidant and linear regression studies was carried out by using Microsoft Excel version 20.0 (The Microsoft Corporation, Redmond, Washington, USA).

## Results and discussion

### Phytochemical profiling

#### The yield of extract

The yield of ethanolic extract of *Pandanus amaryllifolius* Roxb. was 16.24 g. According to preliminary phytochemical analysis, ethanol extract *P*. *amaryllifolius* Roxb. leaves contained high amounts of flavonoid and polyphenols, and moderate level of alkaloid and terpenoids. However, saponin was not detected in the ethanol extract as shown in S1 Table. Flavonoids and polyphenols are well known for their antioxidant and anti-inflammatory properties [32]. Alkaloids and terpenoids have also been shown to have several biological activities including anti-inflammatory, anticancer, and antimicrobial properties [33]. The absence of saponins in the ethanol extract is an indication that it may not have any cholesterol-lowering properties [34]. The GC-MS analysis was carried out for the identification of the specific compounds that responsible for antimicrobial properties of the extract.

#### Gas chromatography-mass spectrometry (GC-MS) analysis

**Fig 1:**
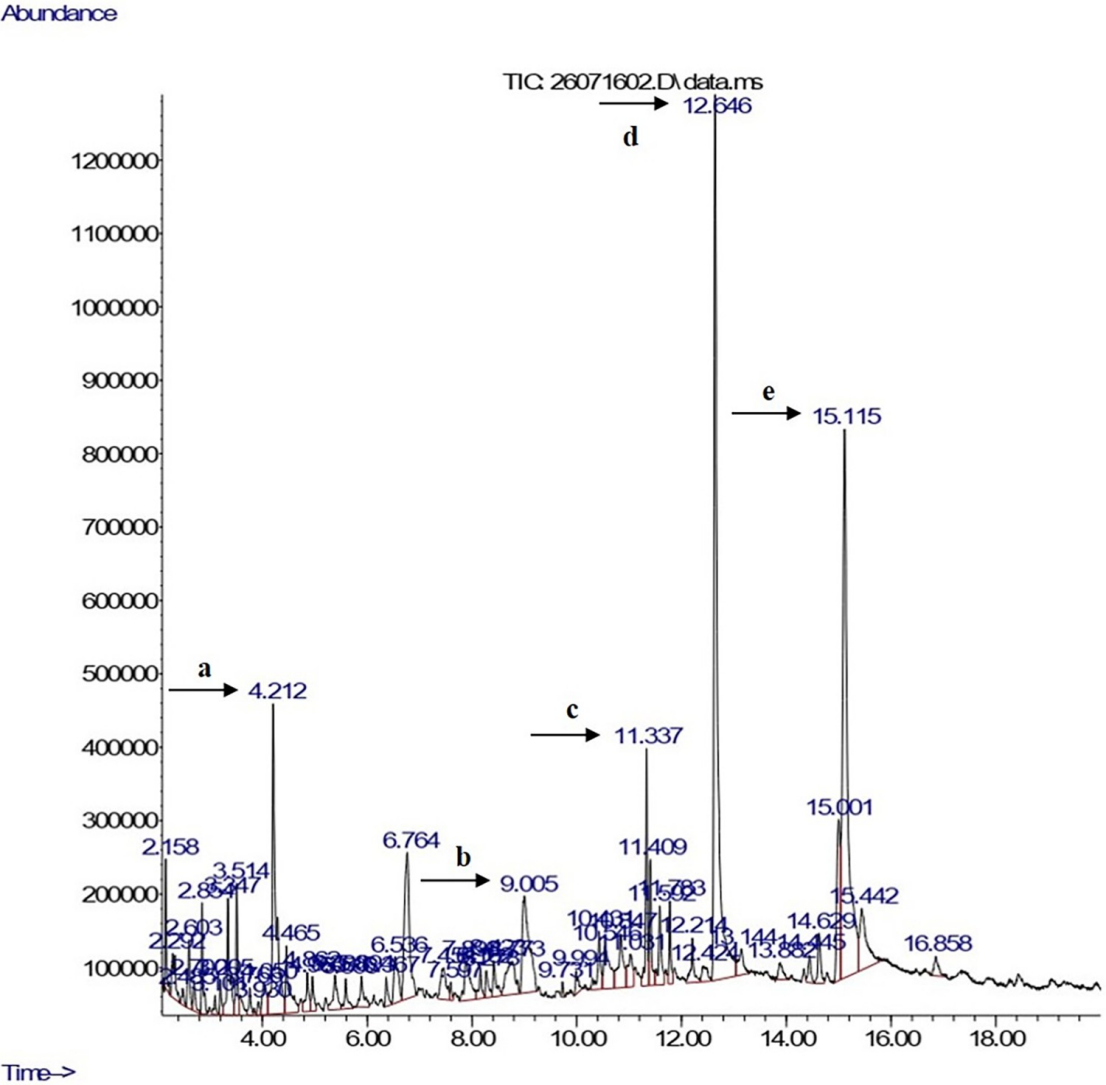
The gas chromatogram of the constituent compounds from the ethanol extract of *Pandanus amaryllifolius* Roxb. leaves. Numbered arrow showed the major compound of the extract with (a) benzofuran 2,3-dihydro-; (b) quinic acid; (c) neophytadiene; (d) *n*-hexadecanoic acid; (e) 9, 12, 15-octadecatrienoic acid.

The gas chromatogram of the constituent compounds from the ethanol extract is shown in Fig 1. The GC-MS analysis strongly supported to find 52 bioactive compounds. The active principles with their respective retention time (RT) and concentration (peak area %) are given in S2 Table. The chromatogram showed that compound contained *n*-hexadecanoic acid (19.31%); 9,12,15-octadecatrienoic acid (17.82%); and benzofuran, 2,3-dihydro- (6.84%) as major compound. Therefore, the compounds were shown to have antimicrobial activity based on the references [2, 3].

### *In silico* antimicrobial activity

#### Ligand retrieval and protein preparation

**Fig 2:**
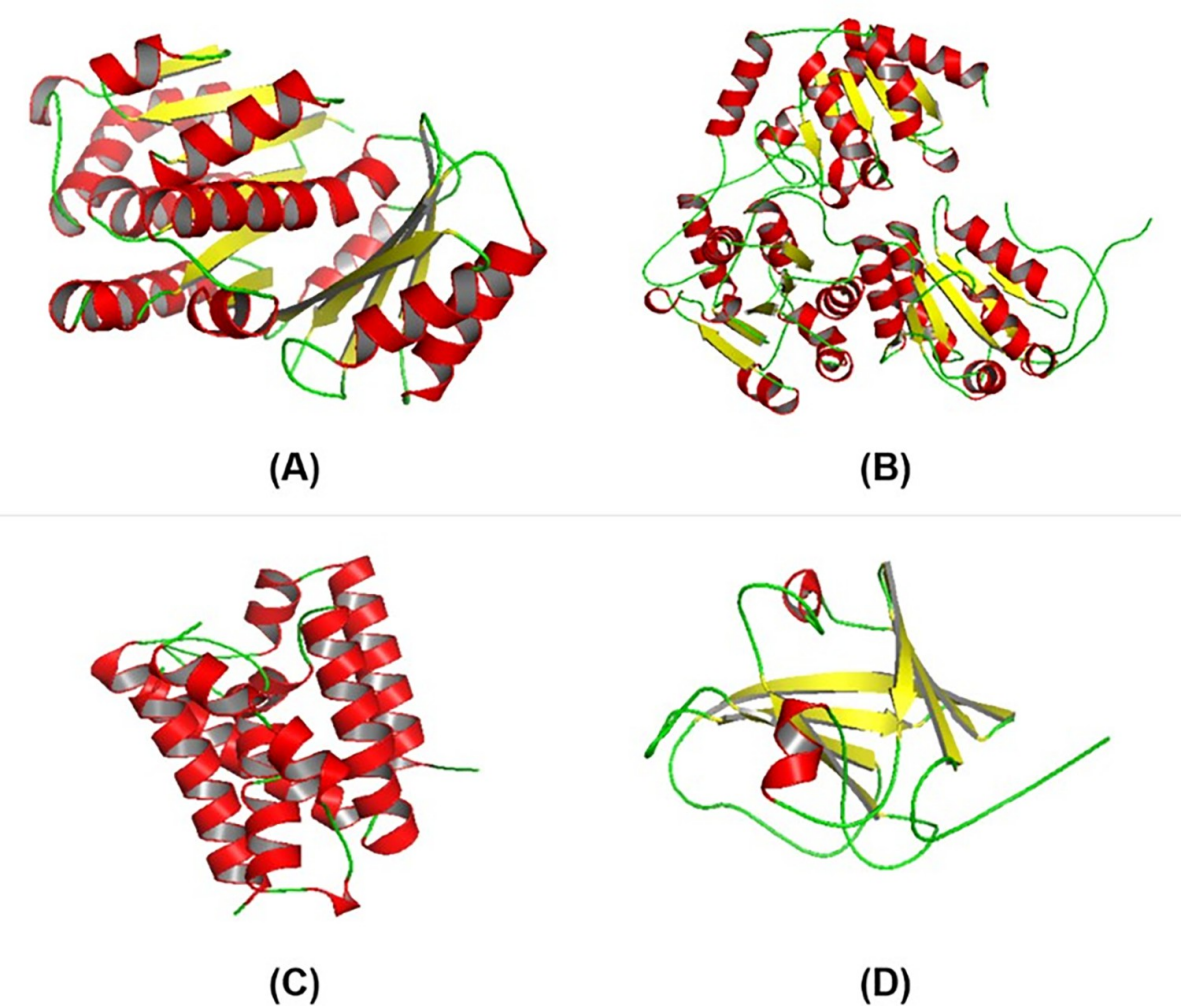
Antimicrobial targets of microorganisms consisting of *Bacillus subtilis*, *Candida albicans*, *Escherichia coli*, and *Staphylococcus aureus*. (A) FtsZ; (B) AHAS; (C) Rpro; (D) SA. Secondary protein structures such as the α-helix (red), β-sheet (yellow), and coil (green).

The compounds from *P*. *amaryllifolius* Roxb. were attained from the PubChem database (https://pubchem.ncbi.nlm.nih.gov/), which provides information consisting of compound name, Collision-induced dissociation (CID), formula, structure data format (sdf) file, and SMILE Canonical in Table 1. SMILE Canonical and sdf file were used as input data for identifying drug-like molecules from *P*. *amaryllifolius* Roxb. through Lipinski’s Rule of Five. The protein samples used in this study consisted of FtsZ—*Bacillus subtilis* (RCSB ID: 2VAM), acetohydroxyacid synthase (AHAS)—*Candida albicans* (RCSB ID: 6DEK), rhomboid protease (Rpro)—*Escherichia coli* (RCSB ID: 3ZMI), and Sortase A (SA)—*Staphylococcus aureus* (RCSB ID: 2MLM), which were obtained from RCSB (http://www.rcsb.org/pdb/home/home.do). *Bacillus subtilis* uses the protein FTsZ to regulate the cell division process, while the AHAS enzyme in *Candida albicans* plays a role in the virulence, invasion, and pathogenicity of the fungus [35, 36]. The activity of Rpro in *Escherichia coli* and SA in *Staphylococcus aureus* as targets for antibiotic binding is essential in the mechanism of bacterial infection in humans [37]. The 3D structure visualization of the targets, consisting of FtsZ, AHAS, Rpro, and SA, was done through PyMol v2.5 using coloring by secondary structure (Fig 2).

**Table 1:** Ligand preparation and information of *P*. *amaryllifolius* Roxb. compounds from PubChem database.

No	Compound	CID	Formula	SMILE
1.	1,2,3-Propanetriol	753	C3H10O4	C(C(CO)O)O
2.	2(5H)-Furanone, 3-methyl-	30945	C5H6O2	CC1 = CCOC1 = O
3.	1H-Azepin-1-amine, hexahydro-	22198	C6H14N2	C1CCCN(CC1)N
4.	Benzyl Alcohol	244	C7H8O	C1 = CC = C(C = C1)CO
5.	3(2H)-Furanone, dihydro-5-isopropy	546095	C7H12O2	CC(C)C1CC (= O)CO1
6.	Uracil, 1-N-methyl	12009	C5H6N2O2	CN1C = CC (= O)NC1 = O
7.	1,3-Heptadiene, 2,3-dimethyl-	5370123	C9H16	CCCC = C(C)C (= C)C
8.	3-Ethyl-3-heptanol	88241	C9H20O	CCCCC(CC)(CC)O
9.	N-Met Ethanol, 2-amino-	700	C2H7NO	C(CO)N
10.	4H-Pyran-4-one, 2,3-dihydro-3,5-dihydroxy-6-methyl-	119838	C6H8O4	CC1 = C(C (= O)C(CO1)O)O
11.	-Pyrrolidinepropanol,.alpha.-cyclohexyl-.alpha.-phenyl-	207840	C19H29NO	C1CCC(CC1)C(CCN2CCCC2)(C3 = CC = CC = C3)O.Cl
12.	Furan, 2-ethoxy-2,3-dihydro-4-methyl-	551516	C7H12O2	CCOC1CC (= CO1)C
13.	Phenol, 2-ethoxy-	66755	C8H10O2	CCOC1 = CC = CC = C1O
14.	Benzofuran, 2,3-dihydro-	10329	C8H8O	C1COC2 = CC = CC = C21
15.	Benzonitrile	7505	C7H5N	C1 = CC = C(C = C1)C#N
16.	(2,5-Dichlorophenyl) hydrazine	9366	C6H6Cl2N2	C1 = CC (= C(C = C1Cl)NN)Cl
17.	2-(1-Methylideneethyl) cyclopentane-1-carboxaldehyde Dimethyl Acetal isomer	15178998	C11H20O2	CCOC (= O)C1 = C(CC1)C
18.	2H-Pyran, tetrahydro-2-methoxy-	23057	C6H12O2	COC1CCCCO1
19.	2-Cyanobenzaldehyde	101209	C8H5NO	C1 = CC = C(C (= C1)C = O)C#N
20.	Cyclohexanone, oxime	7517	C6H11NO	C1CCC (= NO)CC1
21.	2(1H)-Pyrimidinethione, 4,5-diamino-	3036166	C4H6N4S	C1 = NC (= S)NC (= C1N)N
22.	5-Nonanol, 5-methyl-	141860	C10H22O	CCCCC(C)(CCCC)O
23.	4-amino-1-.beta.-D-ribofuranosyl-2(1H)-pyrimidinone	6175	C9H13N3O5	C1 = CN(C (= O)N = C1N)C2C(C(C(O2)CO)O)O
24.	n-Decanoic acid	2969	C10H20O2	CCCCCCCCCC (= O)O
25.	Thieno(3,2-d)isothiazole	14024710	C5H3NS2	C1 = CSC2 = C1NC (= O)S2
26.	4-Cyclopropylmethylbenzonitrile	55282712	C11H12N2	C1CC1C(C2 = CC = C(C = C2)C#N)N
27.	2(4H)-Benzofuranone, 5,6,7,7a-tetrahydro-4,4,7a-trimethyl-	14334	C11H16O2	CC1(CC(CC2(C1 = CC (= O)O2)C)O)C
28.	Dodecanoic acid	3893	C12H24O2	CCCCCCCCCCCC (= O)O
29.	2,6-Dimethyl-3-(methoxymethyl)-p-benzoquinone	6430513	C10H12O3	CC1 = CC (= O)C (= C(C1 = O)C)COC
30.	1,7-Azuloquinone	9231	C10H6O2	C1 = CC = C2C = CC = C2C = C1
31.	Quinic acid	6508	C7H12O6	C1C(C(C(CC1(C (= O)O)O)O)O)O
32.	Patchoulialcohol	10955174	C15H26O	CC1CCC2(C(C3CCC2(C1C3)C)(C)C)O
33.	Cyclohexanone, 3-(3-butenyl)-	566226	C10H16O	C = CCCC1CCCC (= O)C1
34.	4-((1E)-3-Hydroxy-1-propenyl)-2-methoxyphenol	91753526	C10H12O3	CC = C(C1 = CC (= C(C = C1)O)OC)O
35.	Tetradecanoic acid	11005	C14H28O2	CCCCCCCCCCCCCC (= O)O
36.	(-)-Loliolide	100332	C11H16O3	CC1(CC(CC2(C1 = CC (= O)O2)C)O)C
37.	i-Inositol	892	C6H12O6	C1(C(C(C(C(C1O)O)O)O)O)O
38.	Neophytadiene	10446	C20H38	CC(C)CCCC(C)CCCC(C)CCCC (= C)C = C
39.	2-Pentadecanone, 6,10,14-trimethyl	10408	C18H36O	CC(C)CCCC(C)CCCC(C)CCCC (= O)C
40.	Pentadecylic acid	13849	C15H30O2	CCCCCCCCCCCCCCC (= O)O
41.	2-Hexadecen-1-ol, 3,7,11,15-tetramethyl-, [R-[R*,R*-(E)]]-	5366244	C20H40O	CC(C)CCCC(C)CCCC(C)CCCC (= CCO)C
42.	Hexadecanoic acid, methyl ester	8181	C17H34O2	CCCCCCCCCCCCCCCC (= O)OC
43.	Spiro[3.5]nonan-1-one, 5-methyl-, trans-	557033	C10H16O	CC1CCCCC12CCC2 = O
44.	n-Hexadecanoic acid	985	C16H32O2	CCCCCCCCCCCCCCCC (= O)O
45.	Phosphetane, 1-chloro-2,2,3,4,4-pentamethyl-	549908	C8H16PCl	CC1C(P (= O)(C1(C)C)Cl)(C)C
46.	Heptadecanoic acid	10465	C17H34O2	CCCCCCCCCCCCCCCCC (= O)O
47.	7,10,13-Hexadecatrienoic acid, methyl ester	5367325	C17H28O2	CCC = CCC = CCC = CCCCCCC (= O)OC
48.	Phytol	5280435	C20H40O	CC(C)CCCC(C)CCCC(C)CCCC (= CCO)C
49.	9,12-Octadecadienoic acid (Z,Z)-	3931	C18H32O2	CCCCCC = CCC = CCCCCCCCC (= O)O
50.	9,12,15-Octadecatrienoic acid	860	C18H30O2	CCC = CCC = CCC = CCCCCCCCC (= O)O
51.	Octadecanoic acid	8158	C18H36O2	CCCCCCCCC (= O)O
52.	(1S,15S)-Bicyclo[13.1.0]hexadecan- 2-one	13760785	C16H28O	C1CCCCCCC (= O)C2CC2CCCCC1

#### Drug likeness prediction

Drug-like molecule analysis refers to an evaluation, characterization of a potential drug candidate molecule, and determination of the physicochemical properties of the molecule such as lipophilicity, bioavailability, and stability [38, 39]. This analysis is important for the drug development process because it helps determine the potential of a molecule as an effective drug and minimizes the risk of side effects. Drug likeness analysis in this study aims to determine the drug-like molecule of the query compound. Lipinski Rule’s of Five (http://www.scfbio-iitd.res.in/software/drugdesign/lipinski.jsp) is used for drug likeness analysis, this method determines drug like-molecule through parameters consisting of molecular mass (D), high lipophilicity (LogP), hydrogen bond donors-acceptors, and molar refractivity. Molecular mass affects the mobility of a drug molecule with a value of ≤500 D, LogP value must be ≤5 and the number of hydrogen bonds (≤5 donors and ≤10 acceptors) affects the physicochemical activity and absorption of drug molecules. The activity of drug molecules is also affected by molar refractivity which refers to the ability to induce charge mobility in the target domain with a value of 40–130. All compounds from *P*. *amaryllifolius* Roxb. leaves extract can act as a drug- like molecule (Table 2). All compounds may trigger specific activities such as inhibition and selective permeable passage to reach the target.

**Table 2:** The result of drug likeness analysis of *Pandanus amaryllifolius* Roxb. compounds.

Compound	CID	Molecular Mass (≤500 D)	LOGP (≤5)	Hydrogen Bond		Molar Refractivity (40–130)	Probable
				Donors (≤5)	Acceptors (≤10)		
1,2,3-Propanetriol	753	92.000	-1.668	3	3	20.178	Drug like-molecule
2(5H)-Furanone, 3-methyl-	30945	98.000	0.489	0	2	24.715	Drug like-molecule
1H-Azepin-1-amine, hexahydro-	22198	114.000	0.736	2	2	34.228	Drug like-molecule
Benzyl Alcohol	244	108.000	1.178	1	1	32.364	Drug like-molecule
3(2H)-Furanone, dihydro-5-isopropy	546095	128.000	1.000	0	2	34.201	Drug like-molecule
Uracil, 1-N-methyl	12009	126.000	-0.318	1	4	30.441	Drug like-molecule
1,3-Heptadiene, 2,3-dimethyl-	5370123	124.000	3.308	0	0	43.478	Drug like-molecule
3-Ethyl-3-heptanol	88241	144.000	2.727	1	1	45.056	Drug like-molecule
N-Met Ethanol, 2-amino-	700	61.000	-1.062	3	2	16.140	Drug like-molecule
4H-Pyran-4-one, 2,3-dihydro-3,5-dihydroxy-6-methyl-	119838	144.000	-0.263	2	4	32.294	Drug like-molecule
-Pyrrolidinepropanol,.alpha.-cyclohexyl-.alpha.-phenyl-	207839	287.000	3.940	1	2	87.204	Drug like-molecule
Furan, 2-ethoxy-2,3-dihydro-4-methyl-	551516	128.000	1.673	0	2	34.872	Drug like-molecule
Phenol, 2-ethoxy-	66755	138.000	1.790	1	2	39.275	Drug like-molecule
Benzofuran, 2,3-dihydro-	10329	120.000	1.621	0	1	35.640	Drug like-molecule
Benzonitrile	7505	103.000	1.558	0	1	31.156	Drug like-molecule
(2,5-Dichlorophenyl)hydrazine	9366	177.000	2.279	3	2	44.272	Drug like-molecule
2-(1-Methylideneethyl)cyclopentane-1-carboxaldehyde Dimethyl Acetal isomer	15178998	140.000	1.659	0	2	38.566	Drug like-molecule
2H-Pyran, tetrahydro-2-methoxy-	23057	116.000	1.159	0	2	30.599	Drug like-molecule
2-Cyanobenzaldehyde	101209	131.000	1.370	0	2	36.544	Drug like-molecule
Cyclohexanone, oxime	7517	113.000	1.679	1	2	32.455	Drug like-molecule
2(1H)-Pyrimidinethione, 4,5-diamino-	3036166	142.000	-0.056	5	3	40.010	Drug like-molecule
5-Nonanol, 5-methyl-	141860	158.000	3.117	1	1	49.673	Drug like-molecule
4-amino-1-.beta.-D-ribofuranosyl-2(1H)-pyrimidinone	6175	243.000	-2.268	5	8	55.757	Drug like-molecule
n-Decanoic acid	2969	172.000	3.211	1	2	50.245	Drug like-molecule
Thieno(3,2-d)isothiazole	14024710	157.000	2.385	1	2	39.194	Drug like-molecule
4-Cyclopropylmethylbenzonitrile	55282712	172.000	1.968	2	2	50.809	Drug like-molecule
2(4H)-Benzofuranone, 5,6,7,7a-tetrahydro-4,4,7a-trimethyl-	14334	196.000	1.409	1	3	51.601	Drug like-molecule
Dodecanoic acid	3893	200.000	3.991	1	2	59.479	Drug like-molecule
2,6-Dimethyl-3-(methoxymethyl)-p-benzoquinone	6430513	180.000	1.047	0	3	48.346	Drug like-molecule
1,7-Azuloquinone	9231	128.000	2.455	0	0	43.412	Drug like-molecule
Quinic acid	6508	192.000	-2.321	5	6	39.839	Drug like-molecule
Patchoulialcohol	10955174	222.000	3.609	1	1	66.066	Drug like-molecule
Cyclohexanone, 3-(3-butenyl)-	566226	152.000	2.711	0	1	46.395	Drug like-molecule
4-((1E)-3-Hydroxy-1-propenyl)-2-methoxyphenol	91753526	180.000	2.319	2	3	50.938	Drug like-molecule
Tetradecanoic acid	11005	228.000	4.772	1	2	68.713	Drug like-molecule
(-)-Loliolide	100332	196.000	1.409	1	3	51.601	Drug like-molecule
i-Inositol	892	180.000	-3.834	6	6	36.040	Drug like-molecule
Neophytadiene	10446	278.000	7.167	0	0	94.055	Drug like-molecule
2-Pentadecanone, 6,10,14-trimethyl	10408	268.000	6.014	0	1	85.399	Drug like-molecule
Pentadecylic acid	13849	242.000	5.162	1	2	73.330	Drug like-molecule
2-Hexadecen-1-ol, 3,7,11,15-tetramethyl-, [R-[R*,R*-(E)]]-	5366244	296.000	6.364	1	1	95.561	Drug like-molecule
Hexadecanoic acid, methyl ester	8181	270.000	5.640	0	2	82.327	Drug like-molecule
Spiro[3.5]nonan-1-one, 5-methyl-, trans-	557033	152.000	2.545	0	1	44.305	Drug like-molecule
n-Hexadecanoic acid	985	256.000	5.552	1	2	77.947	Drug like-molecule
Phosphetane, 1-chloro-2,2,3,4,4-pentamethyl-	549908	194.500	3.710	0	1	50.781	Drug like-molecule
Heptadecanoic acid	10465	270.000	5.942	1	2	82.564	Drug like-molecule
7,10,13-Hexadecatrienoic acid, methyl ester	5367325	264.000	4.968	0	2	82.045	Drug like-molecule
Phytol	5280435	296.000	6.364	1	1	95.561	Drug like-molecule
9,12-Octadecadienoic acid (Z,Z)-	3931	280.000	5.884	1	2	86.993	Drug like-molecule
9,12,15-Octadecatrienoic acid	860	278.000	5.660	1	2	86.899	Drug like-molecule
Octadecanoic acid	8158	158.000	2.821	1	2	45.628	Drug like-molecule
(1S,15S)-Bicyclo[13.1.0]hexadecan- 2-one	13760785	236.000	4.886	0	1	72.007	Drug like-molecule

#### Virtual screening

**Fig 3:**
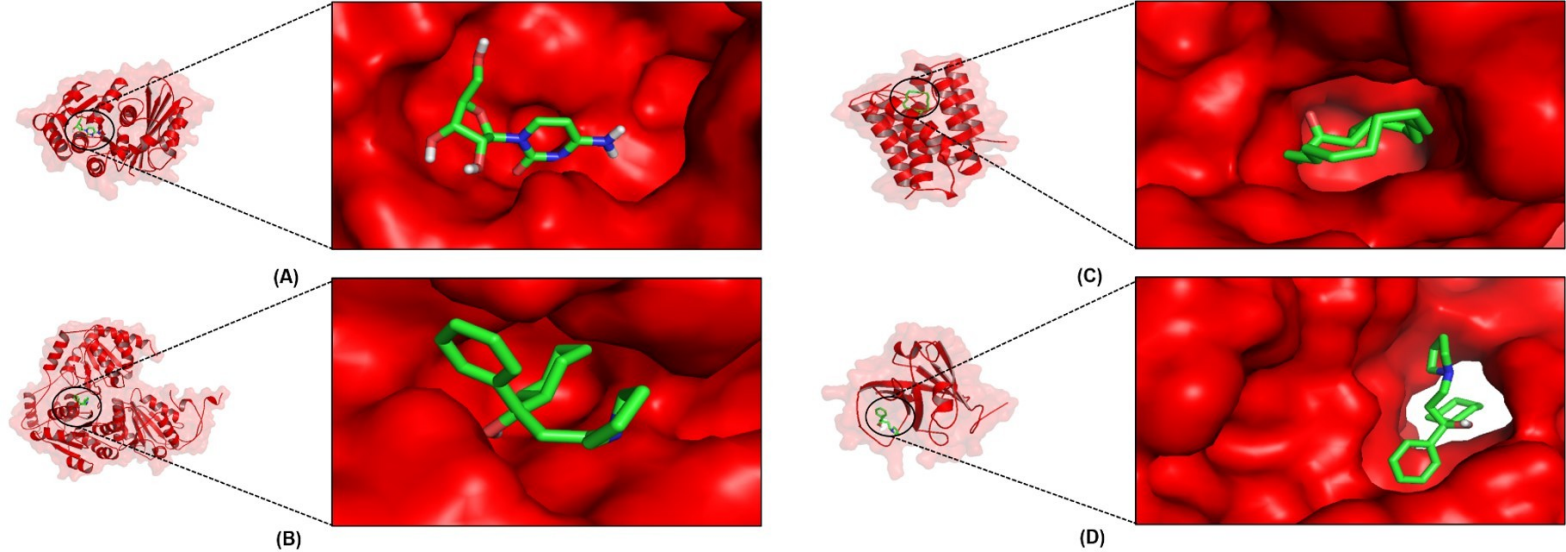
Molecular visualization of the protein-ligand complex. (A) FtsZ_4-amino-1-.beta.-D-ribofuranosyl-2(1H)-pyrimidinone; (B) AHAS_Pyrrolidinepropanol,.alpha.-cyclohexyl-.alpha.-phenyl-; (C) Rpro_(1S,15S)- Bicyclo[13.1.0]hexadecan-2-one; (D) SA_Pyrrolidinepro-panol,.alpha.-cyclohexyl-.alpha.-phenyl.

Virtual screening is the application of computational methods such as molecular docking to determine the activity of natural compounds. Molecular docking plays a role in predicting ligand-target interactions [40]. Ligand activity is determined from energy calculations or binding affinity values to understand the mechanism of this molecular interaction. Binding affinity is a negative bond energy formed due to molecular interactions, the binding affinity value must be negative because it increases the ligand-target interaction strength [41, 42]. Binding affinity is influential in the drug development process because it helps determine the potential of a ligand to become an effective drug. Inhibition of drug molecule activity is determined by the binding affinity value [41]. Docking in this study targets to identify antimicrobial activity of *P*. *amaryllifolius* Roxb. compounds. *P*. *amaryllifolius* Roxb. compounds act as ligands and targets are protein from microbes consisting of *Bacillus subtilis—*FtsZ, *Candida albicans*—AHAS, *Escherichia coli*—Rhomboid Protease (Rpro), and *Staphylococcus aureus*—Sortase A (SA). Compounds from *P*. *amaryllifolius* Roxb. consisting of 4-amino-1-.beta.-D-ribofuranosyl-2(1H)-pyrimidinone (-7.0 kcal/mol—FtsZ—*Bacillus subtilis*), -Pyrrolidinepropanol,.alpha.-cyclohexyl-.alpha.-phenyl—(-7.3 kcal/mol—AHAS—*Candida albicans*, (-7.3 kcal/mol—SA—*Staphylococcus aureus*), and (1S,15S)-Bicyclo[13.1.0]hexadecan-2-one (-8.1 kcal/mol—Rpro—*Escherichia coli*) (Table 3) has a more negative binding affinity than other compounds. These three compounds have the potential as inhibitors of protein activity in microbes. Dual inhibitor refers to the activity of a ligand with more negative binding affinity for the two targets [43, 44]. -Pyrrolidinepropanol, alpha-cyclohexyl-alpha-phenyl- can work through a dual inhibitor mechanism because it has a more negative binding affinity on the two targets. Target inhibition by *Pandanus* sp. compounds triggers inhibition of replication, reproduction, virulence, and invasion of microbes. The 3D structures of candidate antimicrobial compounds are shown through transparent surfaces and sticks with a single-color selection (Fig 3).

**Table 3:** Binding affinity comparison of *Pandanus amaryllifolius* Roxb. compounds.

Compound	CID	Binding Affinity			
		(kcal/mol)			
		FtsZ	AHAS	Rpro	SA
1,2,3-Propanetriol	753	-4.3	-3.9	-4.1	-3.7
2(5H)-Furanone, 3-methyl-	30945	-4.9	-5.2	-4.7	-4.5
1H-Azepin-1-amine, hexahydro-	22198	-5.2	-5.1	-4.9	-4.3
Benzyl Alcohol	244	-5.4	-5.4	-5.4	-3.8
3(2H)-Furanone, dihydro-5-isopropyl	546095	-5.2	-5.6	-5.6	-4.6
Uracil, 1-N-methyl	12009	-5.0	-5.7	-5.7	-5.1
1,3-Heptadiene, 2,3-dimethyl-	5370123	-5.1	-5.0	-5.7	-4.5
3-Ethyl-3-heptanol	88241	-4.4	-5.2	-5.3	-4.2
N-Met Ethanol, 2-amino-	700	-3.3	-3.4	-3.0	-3.0
4H-Pyran-4-one, 2,3-dihydro-3,5-dihydroxy-6-methyl-	119838	-5.4	-5.5	-5.7	-4.8
-Pyrrolidinepropanol,.alpha.-cyclohexyl-.alpha.-phenyl-	207840	-6.5	**-7.3**	**-7.9**	**-7.3**
Furan, 2-ethoxy-2,3-dihydro-4-methyl-	551516	-4.7	-6.6	-5.2	-4.6
Phenol, 2-ethoxy-	66755	-5.3	-5.7	-5.7	-4.7
Benzofuran, 2,3-dihydro-	10329	-5.9	-5.7	-5.9	-5.0
Benzonitrile	7505	-5.4	-5.6	-5.5	-4.8
(2,5-Dichlorophenyl)hydrazine	9366	-5.3	-5.9	-6.1	-4.7
2-(1-Methylideneethyl)cyclopentane-1-carboxaldehyde Dimethyl Acetal isomer	15178998	-5.1	-5.3	-5.7	-4.3
2H-Pyran, tetrahydro-2-methoxy-	23057	-4.6	-4.7	-4.9	-4.1
2-Cyanobenzaldehyde	101209	-5.5	-6.2	-6.0	-5.1
Cyclohexanone, oxime	7517	-6.0	-5.7	-5.5	-5.1
2(1H)-Pyrimidinethione, 4,5-diamino-	3036166	-5.2	-4.8	-4.9	-4.5
5-Nonanol, 5-methyl-	141860	-4.9	-5.0	-5.6	-4.5
4-amino-1-.beta.-D-ribofuranosyl-2(1H)-pyrimidinone	6175	**-7.0**	-6.4	-6.5	-5.8
n-Decanoic acid	2969	-4.5	-4.5	-5.3	-4.7
Thieno(3,2-d)isothiazole	14024710	-5.0	-5.6	-5.0	-4.8
4-Cyclopropylmethylbenzonitrile	55282712	-5.7	-6.5	-6.9	-5.9
2(4H)-Benzofuranone, 5,6,7,7a-tetrahydro-4,4,7a-trimethyl-	14334	-6.0	-6.8	-7.1	-5.8
Dodecanoic acid	3893	-4.5	-4.9	-5.5	-5.1
2,6-Dimethyl-3-(methoxymethyl)-p-benzoquinone	6430513	-6.1	-6.0	-6.5	-4.9
1,7-Azuloquinone	9231	-6.5	-6.5	-6.9	-5.8
Quinic acid	6508	-5.8	-6.2	-5.7	-5.6
Patchoulialcohol	10955174	-5.9	-6.6	-6.7	-6.8
Cyclohexanone, 3-(3-butenyl)-	566226	-5.0	-6.0	-6.1	-5.1
4-((1E)-3-Hydroxy-1-propenyl)-2-methoxyphenol	91753526	-5.8	-6.4	-6.4	-5.4
Tetradecanoic acid	11005	-4.8	-5.8	-5.3	-4.7
(-)-Loliolide	100332	-6.2	-6.8	-7.1	-5.9
i-Inositol	892	-6.0	-5.6	-6.1	-5.1
Neophytadiene	10446	-5.2	-5.1	-6.2	-5.2
2-Pentadecanone, 6,10,14-trimethyl	10408	-4.8	-4.6	-6.3	-5.5
Pentadecylic acid	13849	-4.4	-4.7	-5.4	-5.0
2-Hexadecen-1-ol, 3,7,11,15-tetramethyl-, [R-[R*,R*-(E)]]-	5366244	-5.3	-5.0	-6.1	-5.5
Hexadecanoic acid, methyl ester	8181	-5.0	-4.7	-5.3	-4.9
Spiro[3.5]nonan-1-one, 5-methyl-, trans-	557033	-4.9	-5.9	-6.4	-5.3
n-Hexadecanoic acid	985	-4.4	-4.8	-5.5	-4.9
Phosphetane, 1-chloro-2,2,3,4,4-pentamethyl-	549908	-4.6	-4.9	-5.1	-4.8
Heptadecanoic acid	10465	-4.5	-4.4	-5.4	-4.9
7,10,13-Hexadecatrienoic acid, methyl ester	5367325	-5.4	-5.5	-5.9	-5.0
Phytol	5280435	-5.1	-5.1	-5.5	-5.4
9,12-Octadecadienoic acid (Z,Z)-	3931	-5.1	-4.6	-5.7	-5.4
9,12,15-Octadecatrienoic acid	860	-5.7	-5.0	-5.6	-5.2
Octadecanoic acid	8158	-4.9	-5.4	-5.4	-5.7
(1S,15S)-Bicyclo[13.1.0]hexadecan- 2-one	13760785	-6.3	-6.8	**-8.1**	-6.6

#### Ligand-protein interaction

**Fig 4:**
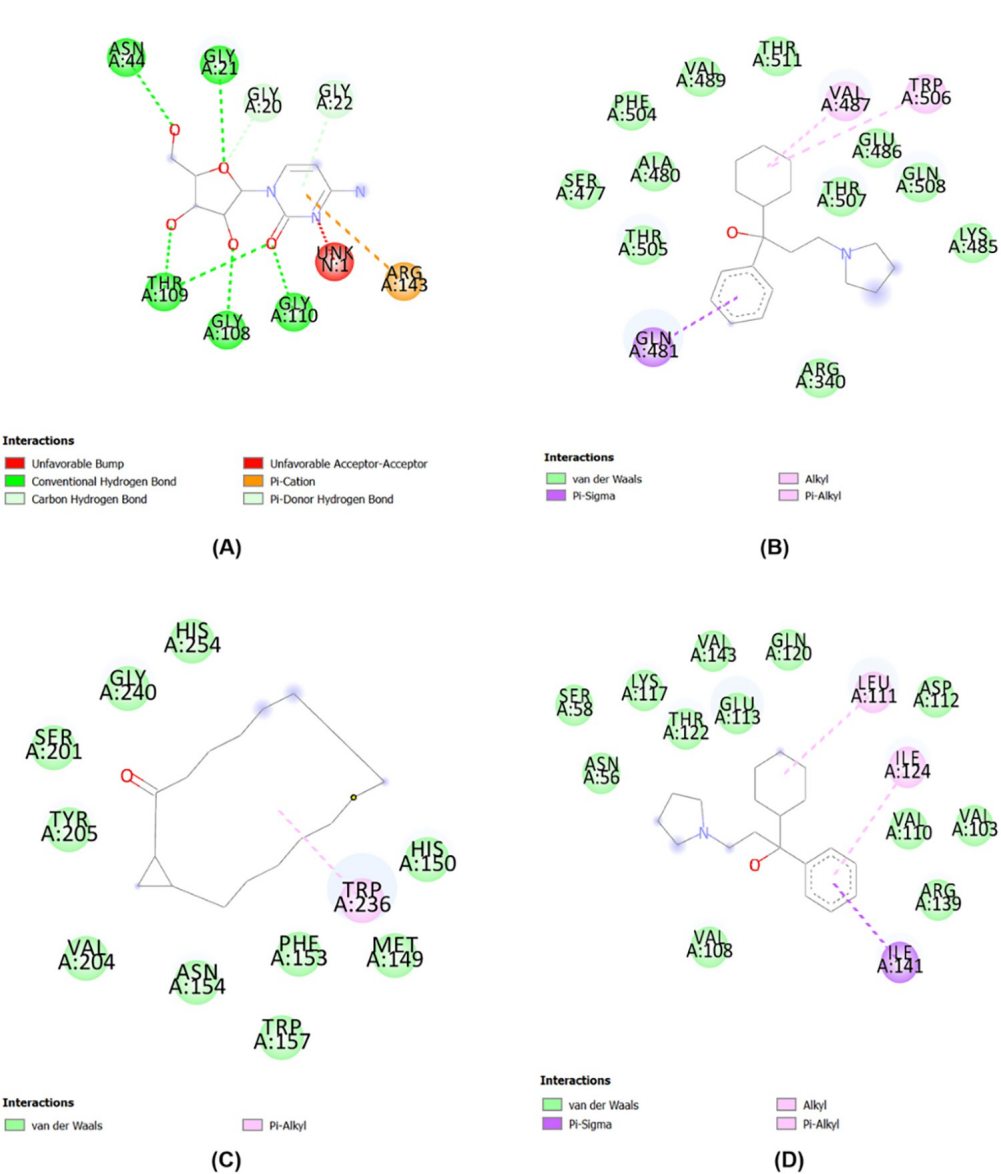
Types of interactions and chemical bond positions. (A) FtsZ_4-amino-1-.beta.-D-ribofuranosyl-2(1H)-pyrimidinone; (B) AHAS_Pyrrolidinepropanol,.alpha.-cyclohexyl-.alpha.-phenyl-; (C) Rpro_(1S,15S)- Bicyclo[13.1.0]hexadecan-2-one; (D) SA_Pyrrolidinepropanol,.alpha.-cyclohexyl-.alpha.-phenyl.

Analysis of molecular interactions in protein-ligand complexes aims to determine the position and type of chemical bonds formed. Weak chemical bonds such as van der Waals, electrostatic, hydrophobic, hydrogen and pi/alkyl bonds are produced by ligands. Weak bonds can trigger ligand activity on targets such as inhibitors. The number of unfavorable interactions on the protein-ligand complex must be <3 to be stable. The results of this study indicate that all ligands can form weak bond interactions (Table 4) such as van der Waals, hydrogen, and Pi/alkyl in the target domain. An unfavorable interaction was formed between 4-amino-1-.beta.-D-ribofuranosyl-2(1H)-pyrimidinone with the FtsZ domain of *Bacillus subtilis*, but does not affect the molecular complex stability (Fig 4). Compounds from *P*. *amaryllifolius* Roxb. consisting of 4-amino-1-.beta.-D-ribofuranosyl-2(1H)-pyrimidinone, Pyrrolidinepropanol,-.alpha.-cyclohexyl-.alpha.-phenyl, and (1S,15S)-Bicyclo[13.1.0] hexadecan-2-one can act as an antimicrobial agent by inhibiting the activity of targets such as FtsZ, AHAS, Rpro, and SA.

**Table 4:** Molecular interaction of protein-ligand based on the binding affinity result.

Microorganism	Protein	Ligand	Chemical Interaction
*Bacillus subtilis*	FtsZ	4-amino-1-.beta.-D-ribofuranosyl-2(1H)-pyrimidinone	Unfavorable: UNKN:1
			van der Waals: Glys20, Gly22
			Hydrogen: Asn44, Thr109, Gly108, Gly110
			Pi: Arg143
*Candida albicans*	AHAS	Pyrrolidinepropanol,.alpha.-cyclohexyl-.alpha.-phenyl-	van der Waals: Ser477, Phe504, Ala480, Thr505, Thr511, Glu486, Thr507, Gln508, Lys485, Arg340
			Pi/Alkyl: Gln481, Val487, Trp506
*Escherichia coli*	Rpro	(1S,15S)-Bicyclo[13.1.0]hexadecan- 2-one	van der Waals: His254, Gly240, Ser201, Tyr205, Val204, Asn154, Trp157, Phe153, Met149, His150
			Pi/Alkyl: Trp236
*Staphylococcus aureus*	SA	Pyrrolidinepropanol,.alpha.-cyclohexyl-.alpha.-phenyl-	van der Waals: Ser58, Lys117, Val143, Gln120, Asn56, Thr122, Glu113, Asp112, Val110, Val103, Arg139, Val108
			Pi/Alkyl: Ile141, Ile124, Leu111

#### Antioxidant activity

DPPH and ABTS assays were carried out to assess the antioxidant activities of the ethanol extract. The IC_50_ values of the extract in comparison with ascorbic acid were presented in Table 5 (S1 Fig). The ethanol extract of *P*. *amaryllifolius* Roxb. possessed high antioxidant activity based on Prieto’s criteria that IC_50_ < 50 μg/ml [45], with IC_50_ value 11.96 ± 4.01μg/ml and 26.18 ± 7.44 μg/ml for DPPH and ABTS assays, respectively. The IC_50_ value of antioxidant activity is a little bit more than ascorbic acid as a positive control. Suwannakul et al. [46] reported DPPH value of IC_50_ of 110.57 ± 36.42 μg/ml for ethanol extract of *Pandanus* sp. Leaves, while Quyen et al. [47] reported IC_50_ 129.327 and 104.31 μg/ml for DPPH and ABTS respectively compared to IC_50_ of 11.96 ± 4.01 μg/ml and 26.18 ± 7.44 μg/ml for DPPH and ABTS obtained from this current study. The observed variations in antioxidants properties are likely due to location of the plant used for these studies.

**Table 5:** *In vitro* antioxidant activity of *Pandanus amaryllifolius* Roxb. leaves ethanol extract.

Sample	Antioxidant activity, IC_50_ (μg/ml)	
	DPPH	ABTS
*P*. *amaryllifolius* Roxb.	11.96 ± 4.01	26.18 ± 7.44
Ascorbic acid	11.42 ± 0.32	12.77 ± 1.30

Note: The data were represented as mean ± SD, n = 3

Moreover, compared to the other studies, the leaf extract from *P*. *amaryllifolius* Roxb. was lower than *Pterocarpus macrocarpus* Kurz. bark extract [3], *Trifolium pratense* L. [48], *Callisia fragrance* leaf juice [45], and *Centella asiatica* L. leaf [49] that have been previously reported as high antioxidant compound. In addition, the antioxidant activity of this study was higher than *Sonchus arvensis* L. [2, 15]. The potent antioxidant activity of the *P*. *amaryllifolius* Roxb. extract was probably due to the presence of active ingredients with antioxidant activities.

#### Antimicrobial activity

**Fig 5:**
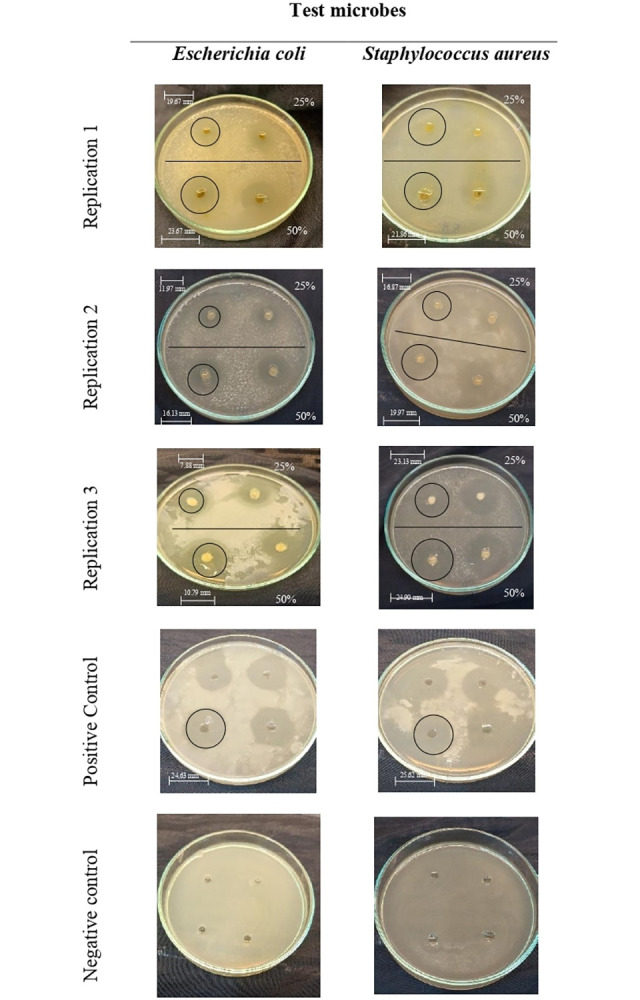
Antimicrobial activity evaluated using agar well diffusion method against *Escherichia coli* and *Staphylococcus aureus*.

*Staphylococcus aureus* and *Escherichia coli* collected in Surabaya were mainly used in this study as representatives of pathogenic bacteria and yeast as representatives of fungi for the analysis of antimicrobial activity. Antimicrobial tests were carried out with all the extracts against bacteria (Table 6, Fig 5), the ethanol extract has antimicrobial activities against all the pathogens. The diameter of inhibition zone (DIZ) and percentage of inhibition were varied for every single pathogen, 13.88 ± 0.48 mm/56.19 ± 2.62% (25%); 16.44 ± 1.21mm/66.76 ± 4.92 (50%), and 15.49 ± 1.00mm/60.05 ± 1.45% (25%); 21.22 ± 0.11mm/82.49 ± 3.91% (50%) for *S*. *aureus* and *E*. *coli* respectively. The results from the antimicrobial analysis formed the basis on which subsequent studies were carried out with the use of only ethanol extract.

**Table 6:** Diameter of inhibition zone and percentage of inhibition of *Pandanus amaryllifolius* Roxb. leaves extracts.

Natural Products	*Staphylococcus aureus*				*Escherichia coli*			
	*25%*		*50%*		*25%*		*50%*	
	DIZ (mm)	PI (%)	DIZ (mm)	PI (%)	DIZ (mm)	PI (%)	DIZ (mm)	PI (%)
*P*. *amaryllifolius* Roxb.	13.88 ± 0.48	56.19 ± 2.62	16.44 ± 1.21	66.76 ± 4.92	15.49 ± 1.00	60.05 ± 1.45	21.22 ± 0.11	82.49 ± 3.91
Chloramphenicol	25.62 ± 0.28				24.63 ± 0.80			

Note: The data are represented as mean ± SD, n = 3. DIZ: diameter of inhibition zone (mm); PI: percentage of inhibition (%); positive control: Chloramphenicol and Nystatin.

Gonelimali et al. (2018) studied antimicrobial property of ethanolic extracts of *Hibiscus sabdariffa* (roselle), *Syzygium aromaticum* (clove), *Rosmarinus officinalis* (rosemary) and *Thymus vulgaris* (thyme) against several food pathogens and food spoiling bacteria. They found zone of inhibition (in mm) 21.1±1.3 (roselle), 17.4±0.8 (rosemary), 21.1±0.9 (clove), 15.9±0.3(thyme) for *E*. *coli* bacteria. However, 21.5±2.1 (roselle), 16.7±1.0 (rosemary), 19.8±0.4 (clove), 16.3±1 (thyme) for *S*. *aureus* [50]. Razmavar et al. (2014) observed antimicrobial activity of ethanolic extracts of *Baeckea frutescens* leaves against *E*. *coli and S*. *aureus* bacteria. They observed inhibition zone (in mm) are of 7 for 20% and 7.5 for 50% against *E*. *coli* and that of 7.5 for 20% and 11.5 for 50% for *S*. *aureus* respectively [51]. Based on these values we can say that our results are in line with previous literature. Hence, *P*. *amaryllifolius* Roxb. possesses potential of antimicrobial activity.

Microbial infection will increase free radicals (reactive oxygen intermediates/ROI, reactive oxygen species/ROS, and nitric oxide synthetize/NO). Free radicals are molecules with one unpaired electron in their outer orbit which makes the molecule unstable [52]. Free radicals can cause oxidative stress. It has implications for various pathological conditions [53]. The involvement of oxidative stress can cause the amount of antioxidant status to decrease [52]. Oxidative stress condition is defined as an imbalance condition between antioxidants and free radicals, where the state of free radicals is higher than antioxidants [52]. The number of antioxidants decreases because the body used to balance the high free radicals due to the presence of parasites. The more severe the infection from microbe, the use of antioxidants in the body will increase, causing the number of antioxidants in the body to decrease [52]. It is very valuable for the further investigation of efficacious the *P*. *amaryllifolius* Roxb. leaf as antimicrobial agent candidate.

The *in silico* molecular docking studies supported the experimental findings and provided insight into the mechanism of action of the bioactive compounds. The results showed that the compounds in *P*. *amaryllifolius* Roxb. extract can effectively bind to the target proteins of the selected pathogens, inhibiting their growth and replication. The *in silico* studies revealed that the extract can serves as an antimicrobial against *Bacillus subtilis*, *Candida albicans*, *Escherichia coli*, and *Staphylococcus aureus* by inhibiting the activity of FtsZ, AHAS, Rpro, and SA via 4-amino-1-.beta.-D-ribofuranosyl-2(1H)-pyrimidinone, -Pyrrolidinepropanol,.alpha.-cyclohexyl-.alpha.-phenyl, and (1S,15S)-Bicyclo[13.1.0]hexadecan-2-one with more negative binding affinity and form stable interactions. The results of this study also revealed that -Pyrrolidinepropanol, alpha.-cyclohexyl-.alpha.-phenyl has dual inhibitory activity on AHAS and SA. Overall, this study provides compelling evidence that *P*. *amaryllifolius* Roxb. leaves is a promising candidate for the expansion of new antimicrobial agents.

## Conclusion

This study presents a wide-raging exploration of the antimicrobial potential of *Pandanus amaryllifolius* Roxb. phytochemical screening uncovered the existence of several bioactive compounds in the plant extract, which may contribute to its antimicrobial activity. Additionally, antioxidant assays demonstrated the plant’s potential to scavenge free radicals, which may further enhance its therapeutic properties. These findings are a significant step forward in the search for novel and effective alternatives to conventional antimicrobial agents.

## Supporting information

S1 Fig Figure of regression linear for DPPH and ABTS result of *Pandanus amaryllifolius* Roxb. leaves extract. Rep: replication.
(DOCX)

S1 Table Preliminary phytochemical screening of *Pandanus amaryllifolius* Roxb. leaves ethanol extracts. (DOCX)

S2 Table GC-MS spectral analysis of ethanol extract of *Pandanus amaryllifolius* Roxb. (DOCX)

S3 Table Antimicrobial result of *Pandanus amaryllifolius* Roxb. leaves extracts. (DOCX)
