# Supplementary material for: Antimicrobial potentials of Pandanus amaryllifolius Roxb.: Phytochemical profiling, antioxidant, and molecular docking studies
Source: PLoS One. 2024 Aug 14;19(8):e0305348. doi: 10.1371/journal.pone.0305348 (PMC11324095; doi:10.1371/journal.pone.0305348)
Supplement: S1 Fig — Rep: replication. (DOCX) [file pone.0305348.s001.docx]

**S3 Fig. Figure of regression linear for DPPH and ABTS result of *Pandanus amaryllifolius* Roxb. leaves extract.** Rep: replication
