# Supplementary material for: Antimicrobial potentials of Pandanus amaryllifolius Roxb.: Phytochemical profiling, antioxidant, and molecular docking studies
Source: PLoS One. 2024 Aug 14;19(8):e0305348. doi: 10.1371/journal.pone.0305348 (PMC11324095; doi:10.1371/journal.pone.0305348)
Supplement: S1 Table — (DOCX) [file pone.0305348.s002.docx]

**S1 Table.** **Preliminary phytochemical screening of *Pandanus amaryllifolius* Roxb. leaves ethanol extracts**

| **No.** | **Phytochemicals** | **Methanolic extract of *Pandanus amaryllifolius* Roxb. Leaves** |
| --- | --- | --- |
| 1 | Terpenoids | ++ |
| 2 | Flavonoids | +++ |
| 3 | Alkaloids | ++ |
| 4 | Saponin | - |
| 5 | Polyphenol | +++ |

*Note: + +, Strongly positive; +, Weakly positive; −, Not detected
