# Supplementary material for: Antimicrobial potentials of Pandanus amaryllifolius Roxb.: Phytochemical profiling, antioxidant, and molecular docking studies
Source: PLoS One. 2024 Aug 14;19(8):e0305348. doi: 10.1371/journal.pone.0305348 (PMC11324095; doi:10.1371/journal.pone.0305348)
Supplement: S2 Table — (DOCX) [file pone.0305348.s003.docx]

**S2 Table. GC-MS spectral analysis of ethanol extract of *Pandanus* *amaryllifolius*** [**Roxb.**](https://id.wikipedia.org/wiki/William_Roxburgh)

| **Peak** | **R.T** | **Compound** | **Molecular formula** | **Molecular weight** | **Area (%)** |
| --- | --- | --- | --- | --- | --- |
| 1 | 2.156 | 1,2,3-Propanetriol | **C_3_H_10_O_4_** | 110.11 | 1.07 |
| 2 | 2.293 | 3-Methyl-2(5H)-furanone | C_5_H_6_O_2_ | 98.0999 | 1.05 |
| 3 | 2.487 | Hexahydro-1H-Azepin-1-amine | C_6_H_14_N_2_ | 114.1888 | 0.24 |
| 4 | 2.602 | Benzyl alcohol | C_7_H_8_O | 108.1378 | 0.82 |
| 5 | 2.699 | Dihydro-5-isopropy-3(2H)-furanone | C_7_H_12_O_2_ | \|  \| 128.17 \| \| --- \| --- \| | 0.46 |
| 6 | 2.853 | 1-N-Methyl-uracil, | C_5_H_6_N_2_O_2_ | \|  \| 126.11 \| \| --- \| --- \| | 1.13 |
| 7 | 3.099 | 2,3-Dimethyl-1,3-heptadiene | C_9_H_16_ | 124.22 | 0.24 |
| 8 | 3.207 | 3-Ethyl-3-heptanol | C_9_H_20_O | 144.2545 | 0.38 |
| 9 | 3.345 | N-Methoxy-methanamine | C_2_H_7_NO | 88.1051 | 2.05 |
| 10 | 3.516 | 2,3-Dihydro-3,5-dihydroxy-6-methyl-4H-pyran-4-one | C_6_H_8_O_4_ | 144.1253 | 1.31 |
| 11 | 3.768 | Alpha-cyclohexyl-alpha-phenyl--pyrrolidinepropanol | C_19_H_29_NO | 287.4397 | 0.37 |
| 12 | 3.928 | 2-Ethoxy-2,3-dihydro-4-methyl-furan | C_7_H_12_O_2_ | 128.17 | 0.19 |
| 13 | 4.048 | 2-Ethoxy-phenol | C_8_H_10_O_2_ | 138.1638 | 0.88 |
| 14 | 4.213 | 2,3-Dihydro-benzofuran, | C_8_H_8_O | 120.1485 | 6.84 |
| 15 | 4.465 | Benzonitrile | C_7_H_5_N | 103.1213 | 1.57 |
| 16 | 4.865 | 2,5-Dichlorophenyl) hydrazine | C_6_H_6_Cl_2_N_2_ | 177.03 | 0.77 |
| 17 | 4.962 | 2-(1-Methylideneethyl) cyclopentane-1-carboxaldehyde Dimethyl Acetal isomer | C_11_H_20_O_2_ | 184.28 | 0.52 |
| 18 | 5.391 | Tetrahydro-2-methoxy-2H-pyran | C_6_H_12_O_2_ | 116.1583 | 0.93 |
| 19 | 5.591 | 2-Cyanobenzaldehyde | C_8_H_5_NO | 131.1314 | 0.55 |
| 20 | 5.894 | Oxime Cyclohexanone | C_6_H_11_NO | 113.1576 | 0.72 |
| 21 | 6.368 | 4,5-Diamino-2(1H)-pyrimidinethione | C_4_H_6_N_4_S | 142.182 | 0.44 |
| 22 | 6.534 | 5-Methyl-5-nonanol | C_10_H_22_O | 158.2811 | 1.33 |
| 23 | 6.762 | 4-amino-1-beta-D-ribofuranosyl-2(1H)-pyrimidinone | C_9_H_13_N_3_O_5_ | 243.2166 | 4.82 |
| 24 | 7.454 | Decanoic acid | C_10_H_20_O_2_ | 172.2646 | 1.12 |
| 25 | 7.597 | Thieno(3,2-d)isothiazole | C_5_H_3_NS_2_ | 141.21 | 0.20 |
| 26 | 7.894 | 4-Cyclopropylmethylbenzonitrile | C_11_H_12_N_2_ | \|  \| 172.23 \| \| --- \| --- \| | 1.53 |
| 27 | 8.163 | 2(4H)-Benzofuranone, 5,6,7,7a-tetrahydro-4,4,7a-trimethyl- | C_11_H_16_O_2_ | 180.2435 | 0.55 |
| 28 | 8.277 | Dodecanoic acid | C_12_H_24_O_2_ | 200.3178 | 0.50 |
| 29 | 8.425 | 2,6-Dimethyl-3-(methoxymethyl)-p-benzoquinone | C_10_H_12_O_3_ | 180.2005 | 0.73 |
| 30 | 8.774 | 1,7-Azuloquinone | C_10_H_6_O_2_ | 158.1534 | 1.94 |
| 31 | 9.003 | Quinic acid | C_7_H_12_O_6_ | 192.17 | 4.33 |
| 32 | 9.729 | Patchouli alcohol | C_15_H_26_O | 222.37 | 0.15 |
| 33 | 9.992 | 3-(3-Butenyl)-cyclohexanone, | C_10_H_16_O | 152.23 | 0.39 |
| 34 | 10.432 | 4-((1E)-3-Hydroxy-1-propenyl)-2-methoxyphenol | C_10_H_12_O_3_ | 180.2005 | 1.15 |
| 35 | 10.546 | Tetradecanoic acid | C_14_H_28_O_2_ | 228.3709 | 1.69 |
| 36 | 10.849 | (-)-Loliolide | C_11_H_16_O_3_ | 196.24 | 1.76 |
| 37 | 11.032 | I-Inositols | C_6_H_12_O_6_ | 180.1559 | 1.02 |
| 38 | 11.335 | Neophytadiene | C_20_H_38_ | 278.5157 | 2.42 |
| 39 | 11.409 | 6,10,14-Trimethyl-2-pentadecanone | C_18_H_36_O | 268.4778 | 1.52 |
| 40 | 11.592 | *n*-Pentadecylic acid | C_15_H_30_O_2_ | 242.3975 | 1.39 |
| 41 | 11.780 | 3,7,11,15-tetramethyl-, [R-[R*,R*-(E)]]-2-Hexadecen-1-ol | C_20_H_40_O | 296.5310 | 0.90 |
| 42 | 12.215 | Hexadecanoic acid, methyl ester | C_17_H_34_O_2_ | 270.4507 | 0.84 |
| 43 | 12.426 | Spiro[3.5]nonan-1-one, 5-methyl-, trans- | C_10_H_16_O | 152.23 | 0.56 |
| 44 | 12.643 | n-Hexadecanoic acid | C_16_H_32_O_2_ | 256.4241 | 19.31 |
| 45 | 13.146 | Phosphetane, 1-chloro-2,2,3,4,4-pentamethyl- | C_8_H_16_PCl | \|  \| 194.64 \| \| --- \| --- \| | 0.90 |
| 46 | 13.884 | Heptadecanoic acid | C_17_H_34_O_2_ | 270.4507 | 0.49 |
| 47 | 14.444 | 7,10,13-Hexadecatrienoic acid, methyl ester | C_17_H_28_O_2_ | 264.4030 | 0.49 |
| 48 | 14.627 | Phytol | C_20_H_40_O | 296.5310 | 0.91 |
| 49 | 14.998 | 9,12-Octadecadienoic acid (Z,Z)- | C_18_H_32_O_2_ | 280.4455 | 3.33 |
| 50 | 15.112 | 9,12,15-Octadecatrienoic acid | C_18_H_30_O_2_ | 278.4296 | 17.82 |
| 51 | 15.444 | Octadecanoic acid | C_18_H_36_O_2_ | 284.4772 | 2.86 |
| 52 | 16.856 | (1S,15S)-Bicyclo[13.1.0]hexadecan- 2-one | C_16_H_28_O | \|  \| 236.39 \| \| --- \| --- \| | 0.55 |
