# Supplementary material for: Antimicrobial potentials of Pandanus amaryllifolius Roxb.: Phytochemical profiling, antioxidant, and molecular docking studies
Source: PLoS One. 2024 Aug 14;19(8):e0305348. doi: 10.1371/journal.pone.0305348 (PMC11324095; doi:10.1371/journal.pone.0305348)
Supplement: S3 Table — (DOCX) [file pone.0305348.s004.docx]

**S3 Table.** **Antimicrobial result of *Pandanus amaryllifolius* Roxb. leaves extracts**

| Sample | | Extract in 25% | | | | | Extract in 50% | | | | |
| --- | --- | --- | --- | --- | --- | --- | --- | --- | --- | --- | --- |
| Microbes | | Rep 1 | Rep 2 | Rep 3 | Mean | SD | Rep 1 | Rep 2 | Rep 3 | Mean | SD |
| *Staphylococcus aureus* | DIZ (mm) | 14.29 | 13.21 | 14.14 | 13.88 | 0.58 | 15.09 | 17.41 | 16.84 | 16.44 | 1.21 |
|  | PI (%) | 58.00 | 53.19 | 57.39 | 56.19 | 2.62 | 61.25 | 70.69 | 68.35 | 66.76 | 4.92 |
| *Escherichia coli* | DIZ (mm) | 15.82 | 15.17 | 15.18 | 15.39 | 0.37 | 21.13 | 20.22 | 22.22 | 21.19 | 1.00 |
|  | PI (%) | 61.72 | 59.19 | 59.23 | 60.05 | 1.45 | 82.46 | 78.90 | 86.71 | 82.69 | 3.91 |

Note: SD: standard deviation, DIZ: diameter of inhibition zone, PI: percentage of inhibition
